# Acquired von Willebrand Syndrome in a Patient Undergoing Extracorporeal Membrane Oxygenation: A Case Report

**DOI:** 10.7759/cureus.42305

**Published:** 2023-07-22

**Authors:** Asmae Ettamri Ouaaba, Raquel Behar Lagares

**Affiliations:** 1 Laboratory Medicine, University Hospital October 12, Madrid, ESP; 2 Biochemistry, Eurofins Megalab, Madrid, ESP; 3 Laboratory Medicine, San Carlos Clinical Hospital, Madrid, ESP

**Keywords:** ecmo, hemostasis, hemorrhage, extracorporeal membrane oxygenation, von willebrand factor, aquired von willebrand syndrome

## Abstract

Acquired von Willebrand syndrome (AvWS) is a rare bleeding disorder caused by dysfunction of the von Willebrand factor (vWF), leading to bleeding manifestations. It usually occurs due to an underlying disorder in patients with no family or personal history of bleeding diathesis. The exact mechanism causing this syndrome is not fully understood, but it involves a complex interplay of factors. Specifically, vWF deficiency or reduced activity can occur due to antibodies, adsorption of vWF onto tumor cells, shear stress, or increased proteolysis. We describe a patient with severe, right-sided heart failure secondary to idiopathic pulmonary hypertension. The patient was admitted to the intensive care unit to be placed on a venoarterial extracorporeal membrane oxygenation (VA ECMO) machine while awaiting bilateral lung transplantation. A few hours after initiation of VA ECMO, the patient experienced epistaxis and continuous bleeding from the cannula tips. The laboratory investigations were based on the measurements of vWF antigen (vWF:Ag), vWF ristocetin cofactor activity (vWF:RCo), and multimer analysis. The obtained results revealed a decreased VWF:RCo/VWF:Ag ratio (<0.7) and the loss of high-molecular-weight multimers of vWF, thus confirming the diagnosis of AvWS. This report reviews how to make the clinical diagnosis of AvWS, including a discussion of necessary laboratory results and their pitfalls, and highlights the importance of having a high index of suspicion of AvWS in the ECMO population so that laboratory values are obtained on time to allow for treatment and successful recovery.

## Introduction

Von Willebrand disease (vWD) is the most common congenital bleeding diathesis. It is associated with mutations in the von Willebrand factor (vWF) gene, resulting in quantitative, structural, and functional anomalies in the vWF protein. Acquired von Willebrand syndrome (AvWS) is a rare bleeding disorder affecting up to 0.04% of the population [1] and occurs most often during adulthood in individuals with no history of bleeding disorders, unlike the hereditary form of vWD. AvWS was first described in 1968 in patients with lupus erythematosus [2], although it has gained more attention in recent years due to its association with cardiovascular disorders, such as congenital heart disease and aortic stenosis. In addition, it has been reported that patients undergoing extracorporeal membrane oxygenation (ECMO) or ventricular assist device (VAD) support may develop AvWS [3] due to the shear stress of blood flow within the extracorporeal circuits. The incidence of AvWS in the ECMO population is remarkably high, with some studies reporting a prevalence of up to 100% [3,4]. The high flows generated in ECMO machines and the change in blood flow velocity can lead to a loss of the high-molecular-weight multimers (HMWMs) of vWF.

## Case presentation

A 46-year-old man was referred to our hospital from another medical facility due to severe, right-sided heart failure that was unresponsive to furosemide diuretic treatment. The patient had a history of idiopathic pulmonary hypertension (IPH) and type II diabetes mellitus. He was admitted to the hospital due to lower limb edema, hepatomegaly, an increased abdominal diameter, weight gain, decreased urine output caused by acute renal dysfunction, and progressive dyspnea that evolved into dyspnea on minimal exertion.

Upon physical examination, the patient was afebrile, exhibiting tachycardia and tachypnea. He had a distended abdomen with a fluid wave suggestive of ascites. Cardiac auscultation revealed a mitral holosystolic murmur with the second heart sound reinforcement. The echocardiogram showed severe right heart dilatation with severe functional tricuspid regurgitation and pulmonary artery pressure of 49 mmHg.

The patient was admitted to the intensive care unit (ICU) for venoarterial ECMO (VA ECMO), resulting in improved lung function, hemodynamic normalization, and resolving liver congestion. He was put on the waiting list for bilateral lung transplantation. A few hours after the initiation of VA ECMO, the patient experienced epistaxis and continuous bleeding from the cannula tips, with anemia requiring transfusion and the discontinuation of heparin anticoagulation.

As presented in Table 1, the initial laboratory analysis showed low platelet counts of 92 × 103/μL (confirmed by subsequent peripheral blood smear tests) and normocytic anemia with hemoglobin levels of 7.5 g/dL. The other complete blood count results showed no significant changes. The activated partial thromboplastin time (APTT) and prothrombin time (PT) were both within normal limits, while the platelet function analyzer (PFA)-100 closure times for collagen-ADP (>207 s) and collagen-epinephrine (>218 s) were prolonged. The fibrinogen concentration was 98 mg/dL. The vWF test revealed low levels of vWF antigen (vWF:Ag, 56%) and vWF ristocetin cofactor (vWF:RCo, 35%). The vWF:RCo/vWF:Ag ratio was low at 0.6. The factor VIII clotting activity (FVIII:C) was 46%, and the factor XIII concentration was 16.60%. The lupus anticoagulant test result was negative. The analysis of vWF multimers showed a loss of the HMWMs.

**Table 1:** Patients’ laboratory findings. vWF:Ag, von Willebrand factor antigen; vWF:RCo, ristocetin cofactor activity; PFA, platelet function analyzer; ADP, adenosine diphosphate

Labs	Value	Reference range
Hemoglobin	7.5	13.5-18.0 g/dL
Platelet	92	130 × 10^3 ^to 450 × 10^3^/μL
PFA-100 collagen-ADP	>207	68-121 s
PFA-100 collagen-epinephrine	>218	84-160 s
Activated partial thromboplastin time	38	20-39 s
Prothrombin time	11	9.4-12 s
International normalized ratio	0.98	0.9-1.1
Fibrinogen	98	196-441 mg/dL
Antithrombin	102	80-120 %
Dilute Russell's viper venom time	<1.20	<1.20
Factor VIII coagulant activity	46	60%-150%
Factor XIII	16.60	59%-151%
vWF:Ag	56	60%-150%
vWF:RCo	35	60%-150%
vWF:RCo/vWF:Ag ratio	0.6	>0.7

Combined with the lack of a family history of bleeding, these findings suggested a diagnosis of AvWS, compensated coagulopathy, thrombopathy, and factor XIII deficiency. These findings were thought to be attributable to the use of ECMO.

## Discussion

vWF is a large glycoprotein that actively participates in primary hemostasis and coagulation, affecting the adhesion of platelets to injured vascular surfaces. It is synthesized in megakaryocytes and endothelial cells and circulates in plasma as a series of dimers of 300 kD and multimers of up to 20,000 kD. The latter plays an important role in platelet aggregation. Large multimers provide such functional activity, while ultra-large multimers possess thrombogenic activity. Initially, vWF contributes to primary hemostasis by binding to the collagen of the damaged vascular subendothelium and glycoprotein Ibα (GPIbα) on the platelet surface, thereby favoring platelet adhesion. Subsequently, platelets bind to vWF via GPIIb/IIIa and collagen via GPIa/IIa and GPVI. vWF promotes platelet-platelet junctions to form the platelet hemostatic plug. vWF also binds to FVIII, increasing its half-life by forming a complex that protects it against early proteolysis by physiological inhibitors, such as activated protein C. Low concentrations of vWF can lead to decreased plasma FVIII levels, causing bleeding that resembles hemophilia A. The half-life of FVIII is reduced by up to sixfold in the absence of vWF [5].

AvWS is a rare bleeding disorder caused by heterogeneous mechanisms, that is likely underdiagnosed and arises from acquired vWF deficiency or dysfunction. It is often associated with various underlying conditions, including lymphoproliferative disorders like multiple myeloma; myeloproliferative disorders, such as polycythemia vera; autoimmune disorders, such as lupus erythematosus; malignant neoplasms; and, less commonly, hypothyroidism, certain medications, and other medical conditions [6].

In the context of lymphoproliferative disorders (monoclonal gammopathy of undetermined significance and multiple myeloma) and autoimmune disorders, AvWS is associated with the presence of circulating antibodies that neutralize functional domains of vWF or form immune complexes with vWF, leading to their rapid clearance from the circulation by the reticuloendothelial system [7]. In myeloproliferative disorders, an elevated platelet count may promote the adsorption of vWF onto the cell membranes, leading to subsequent clearance from the plasma [8]. Increased plasma vWF proteolysis can also cause the syndrome in myeloproliferative disorders. The selective adsorption of HMW multimers on tumor cells has also been described in lymphoproliferative diseases (multiple myeloma, Waldenström's macroglobulinemia, non-Hodgkin lymphoma, and hairy cell leukemia) and solid cancer [7]. In patients with hypothyroidism, the pathophysiological mechanism involves impaired synthesis and release of vWF [9]. AvWS has also been described in patients with cardiovascular diseases, such as aortic stenosis, obstructive hypertrophic cardiomyopathy, and congenital heart disease [10], as well as after the use of mechanical circulatory support systems, such as VAD and ECMO, which generate increased plasma clearance of vWF HMWMs due to the shear stress of the blood flow in the extracorporeal circuits. ECMO-induced aVWS has recently been described in patients with COVID-19 who require ECMO support [11].

ECMO refers to a group of assistive therapies used with increasing frequency to provide cardiopulmonary support to patients with life-threatening cardiorespiratory failure when conventional therapeutic measures have failed. The primary function of ECMO is to provide temporary support, acting as a bridge until a medical decision is made or recovery is achieved. In this case report, ECMO was used as a bridge until a compatible donor for lung transplantation was located. Unfortunately, several complications occur with the use of ECMO devices, including bleeding, the most frequent complication with a reported incidence of 30% to 60% [3]. Mechanisms contributing to ECMO bleeding include impaired platelet function, fibrinolysis, and AvWS. Bleeding occurs mainly in surgical access areas, mucous membranes, and the lungs. Intracranial bleeding occurs in 10% of the patients on ECMO support, increasing the mortality rate to 80% [3]. When subjected to shear stress, such as in ECMO systems, vWF undergoes a conformational change and unfolds, exposing active sites, such as the A2 domain that interacts with the ADAMTS13 protease, a protein mainly synthesized in the liver that cleaves large HMWMs, generating smaller and less hemostatic multimeters. This pathophysiology of AvWS is similar to that of hereditary vWD subtype 2A [12], characterized by genetic mutations in the A1 and A2 domains of vWF, resulting in increased susceptibility to proteolysis by ADAMTS13, decreased binding to platelets via GPIbα, and altered multimeric patterns owing to the absence of HMWMs [13].

AvWS was evaluated as a potential cause for the patient's bleeding. A laboratory diagnosis of AvWS is challenging due to the multifactorial etiology of the disease and the lack of standardization in diagnostic tests. The laboratory tests that can guide the diagnosis include the platelet count, complete blood count, peripheral blood smear, APTT, PT, and PFA-100. A prolonged APTT or PFA-100 demonstrates defects in primary hemostasis. The use of PFA-100 is controversial due to its low specificity and falsely prolonged results in patients with anemia, low platelet count, or low hematocrit [12]. Specific tests include the determination of vWF:Ag, which measures the concentration of vWF in the plasma, and vWF:RCo, which measures the ability of platelets to agglutinate in the presence of the antibiotic ristocetin. The binding capacity of vWF to collagen is assessed in the vWF:CB test. The vWF clotting activity is also measured. Expected abnormalities in the AVWS include a normal or low VWF:Ag level and a normal or low FVIII:C in contrast to the marked decrease in vWF:RCo and VWF:CB. Decreased vWF:RCo/vWF:Ag or vWF:CBA/vWF:Ag ratios (<0.7) are also observed in AvWS. These findings suggest the presence of a dysfunctional vWF, resulting in decreased binding to collagen and platelets [5], and were observed in the patient presented in this case report.

vWF:RCo activity assays are limited by their low accuracies and sensitivities [14]. To help address these limitations, the International Society on Thrombosis and Hemostasis recently published new guidelines recommending alternative automated methods for measuring vWF platelet binding activity. These assays use recombinant GPIbα fragments instead of platelets. Some assays use ristocetin to trigger vWF-GPIb (vWF:GPIbR) binding and others rely on the spontaneous binding of a gain-of-function recombinant GPIbα fragment that can bind to vWF in the absence of ristocetin (vWF:GPIbM). A recent study reported that measuring the vWF:GPIbM/vWF:Ag ratio is promising as it has a high positive predictive value [15].

Multimer analyses should follow the activity and concentration assays. vWF multimer analysis using agarose gel electrophoresis is currently the most sensitive and specific method for diagnosing AvWS. It is possible to visualize the different sizes of the vWF multimers and detect any abnormalities in their distributions [16]. In the current patient, the diagnosis of AvWS was based on the lack of HMWMs and a decreased vWF:RCo/vWF:Ag ratio. An FXIII deficiency was also detected and might have contributed to the patient’s bleeding symptoms. FXIII concentrations <30% increase the risk of bleeding [17].

Despite the recent advances in extracorporeal oxygenation technologies, it remains impossible to control the interaction of blood with the biomaterials lining the ECMO circuits, which are potentially thrombogenic or hemorrhagic. After ECMO explanation, recovery from AvWS occurs rapidly within 3 to 24 h [3]. The patient in this study was administered desmopressin, FVIII/vWF concentrates, fresh-frozen plasma, and platelet concentrates to help control the bleeding. Ultimately, the patient underwent successful bilateral lung transplantation.

## Conclusions

This report describes the case of a patient with right-sided heart failure caused by IPH who developed AvWS after ECMO support. Given the high prevalence of AvWS among patients undergoing ECMO therapy, it is imperative to investigate VWF parameters in patients experiencing bleeding symptoms. This syndrome necessitates close collaboration between physicians and the clinical laboratory to ensure an accurate diagnosis and appropriate patient management. The clinical diagnosis of AvWS can be challenging, requiring precise testing for FVIII, vWF:Ag, and various vWF activity assays, including vWF:CB, vWF:RCo, and HMWM. The introduction of rapid and accurate automated assays should facilitate this process. The management of bleeding episodes entails the administration of multiple hemostatic drugs. However, it is preferable to adopt a proactive coagulation management approach based on parameters associated with acquired coagulation, rather than solely treating the clinical bleeding symptoms.

## References

[1] Kumar S, Pruthi RK, Nichols WL (2002). Acquired von Willebrand disease. Mayo Clin Proc.

[2] Simone JV, Cornet JA, Abildgaard CF (1968). Acquired von Willebrand’s syndrome in systemic lupus erythematosus. Blood.

[3] Kalbhenn J, Schlagenhauf A, Rosenfelder S, Schmutz A, Zieger B (2018). Acquired von Willebrand syndrome and impaired platelet function during venovenous extracorporeal membrane oxygenation: rapid onset and fast recovery. J Heart Lung Transplant.

[4] Kalbhenn J, Schmidt R, Nakamura L, Schelling J, Rosenfelder S, Zieger B (2015). Early diagnosis of acquired von Willebrand Syndrome (AVWS) is elementary for clinical practice in patients treated with ECMO therapy. J Atheroscler Thromb.

[5] Pipe SW, Montgomery RR, Pratt KP, Lenting PJ, Lillicrap D (2016). Life in the shadow of a dominant partner: the FVIII-VWF association and its clinical implications for hemophilia A. Blood.

[6] Franchini M, Lippi G (2007). Recent acquisitions in acquired and congenital von Willebrand disorders. Clin Chim Acta.

[7] Richard C, Cuadrado MA, Prieto M (1990). Acquired von Willebrand disease in multiple myeloma secondary to absorption of von Willebrand factor by plasma cells. Am J Hematol.

[8] Mital A, Prejzner W, Bieniaszewska M, Hellmann A (2015). Prevalence of acquired von Willebrand syndrome during essential thrombocythemia: a retrospective analysis of 170 consecutive patients. Pol Arch Med Wewn.

[9] Bruggers CS, McElligott K, Rallison ML (1994). Acquired von Willebrand disease in twins with autoimmune hypothyroidism: response to desmopressin and L-thyroxine therapy. J Pediatr.

[10] Mehta R, Athar M, Girgis S, Hassan A, Becker RC (2019). Acquired Von Willebrand Syndrome (AVWS) in cardiovascular disease: a state of the art review for clinicians. J Thromb Thrombolysis.

[11] Kalbhenn J, Glonnegger H, Büchsel M, Priebe HJ, Zieger B (2022). Acquired von Willebrand syndrome and desmopressin resistance during venovenous extracorporeal membrane oxygenation in patients with COVID-19: a prospective observational study. Crit Care Med.

[12] Horiuchi H, Doman T, Kokame K, Saiki Y, Matsumoto M (2019). Acquired von Willebrand syndrome associated with cardiovascular diseases. J Atheroscler Thromb.

[13] Castaman G, Linari S (2017). Diagnosis and treatment of von Willebrand disease and rare bleeding disorders. J Clin Med.

[14] Harris NS, Pelletier JP, Marin MJ, Winter WE (2022). Von Willebrand factor and disease: a review for laboratory professionals. Crit Rev Clin Lab Sci.

[15] Icheva V, Ebert J, Budde U (2023). Perioperative diagnosis and impact of acquired von Willebrand syndrome in infants with congenital heart disease. Blood.

[16] Pikta M, Banys V, Szanto T (2021). Von Willebrand factor multimeric assay in acquired Von Willebrand disease diagnosis: a report of experience from North Estonia Medical Centre. J Lab Physicians.

[17] Thomas J, Kostousov V, Teruya J (2018). Bleeding and thrombotic complications in the use of extracorporeal membrane oxygenation. Semin Thromb Hemost.

